# A systematic review on the usability of robotic and virtual reality devices in neuromotor rehabilitation: patients’ and healthcare professionals’ perspective

**DOI:** 10.1186/s12913-022-07821-w

**Published:** 2022-04-20

**Authors:** Francesco Zanatta, Anna Giardini, Antonia Pierobon, Marco D’Addario, Patrizia Steca

**Affiliations:** 1 Department of Psychology, University of Milano-Bicocca, Milan, Italy; 2 Information Technology Department, Istituti Clinici Scientifici Maugeri IRCCS, Pavia, Italy; 3 Psychology Unit of Montescano Institute, Istituti Clinici Scientifici Maugeri IRCCS, Montescano, Italy; 4 Department of Psychology, University of Milano-Bicocca, Milan, Italy

**Keywords:** Virtual Reality, Robotics, Usability, Systematic Review, Rehabilitation

## Abstract

**Background:**

The application of virtual reality (VR) and robotic devices in neuromotor rehabilitation has provided promising evidence in terms of efficacy, so far. Usability evaluations of these technologies have been conducted extensively, but no overviews on this topic have been reported yet.

**Methods:**

A systematic review of the studies on patients' and healthcare professionals' perspective through searching of PubMed, Medline, Scopus, Web of Science, CINAHL, and PsychINFO (2000 to 2021) was conducted. Descriptive data regarding the study design, participants, technological devices, interventions, and quantitative and qualitative usability evaluations were extracted and meta-synthetized.

**Results:**

Sixty-eight studies were included. VR devices were perceived as having good usability and as a tool promoting patients' engagement and motivation during the treatment, as well as providing strong potential for customized rehabilitation sessions. By contrast, they suffered from the effect of learnability and were judged as potentially requiring more mental effort. Robotics implementation received positive feedback along with high satisfaction and perceived safety throughout the treatment. Robot-assisted rehabilitation was considered useful as it supported increased treatment intensity and contributed to improved patients' physical independence and psychosocial well-being. Technical and design-related issues may limit the applicability making the treatment difficult and physically straining. Moreover, cognitive and communication deficits were remarked as potential barriers.

**Conclusions:**

Overall, VR and robotic devices have been perceived usable so far, reflecting good acceptance in neuromotor rehabilitation programs. The limitations raised by the participants should be considered to further improve devices applicability and maximise technological rehabilitation effectiveness.

**Trial registration:**

PROSPERO registration ref. CRD42021224141.

**Supplementary Information:**

The online version contains supplementary material available at 10.1186/s12913-022-07821-w.

## Background

In the last two decades, robotic and virtual reality (VR) devices have gained increased interest in the rehabilitation community for their multipurpose application in patient’s physical recovery process [1, 2]. Robot-assisted rehabilitation has showed promising results so far, thanks to its peculiarity to provide intensive, repetitive and task-oriented activities for the treatment of motor impairment resulting from various neurological and musculoskeletal diseases [3]. Compared to conventional therapies, it benefits from smaller workforce, optimized exercise, and quantitative assessment and monitoring. Moreover, among its advantages are also the possibility to better tailor the interventions, by increasing the amount and quality of the therapy that can be administered, and by managing the parameters to make the rehabilitation personalized to the patient [4]. To date, mainly two different types of robotic devices have been implemented, for both lower and upper limbs rehabilitation. The first is based on the use of exoskeletons, which are systems constituted by mechanical and electronic components that completely cover the limb, following its anthropometric characteristics, and assist the kinematic or dynamic activity that patient performs [5, 6]. The second is of the end-effector type. Differently from the exoskeletons, robotic end-effector devices interconnect to the distal part of the limb, allowing the natural kinematic activation of the movement without specific constraints and, thus, with more degrees of freedom [7, 8]. Additionally, another typology is represented by soft-robotics. Soft-robots are wearable devices characterized by a lightweight and flexible structure, and although they are primarily intended to be worn and used for the support and assistance of the activities of daily living (ADLs), they have also been shown promising tools for rehabilitation purposes [9].

Similarly to robotics, VR is considered an emerging tool in the field of rehabilitation, representing a trending and widely accessible technology for the treatment of different medical conditions [10–12]. It can be defined as a system based on computer-simulated 3D environments allowing the user to navigate through and interact with by the integration of auditory, visual, and haptic feedback [13]. Accordingly, VR has three key characteristics: immersion, presence, and interactivity [14]. Immersion refers to the degree to which VR can provide multisensory stimuli, originating from the virtual environment (VE), and a high degree of matching between user’s actions and the cues generated by the system. Consequently, the immersion in and the interactivity with the VEs affect patient’s experience and perception and, thus, his/her sense of presence [15]. Based on the level of immersion, VR devices and systems can be categorized in fully-immersive, semi-immersive, and non-immersive [16]. Fully-immersive systems are characterized by the use of tools, as an head-mounted display (HMD) or a cave automatic virtual environment (CAVE), that enable a high degree of immersion and interaction with the VE, blocking out patient’s perception of the real-world. Differently, semi-immersive systems provide a moderate level of immersion and interaction and usually consist of large monitors or projectors that let the patient perceive the real-world and a part of the VE, simultaneously. Lastly, non-immersive systems allow for a low immersion and interaction and include simpler devices such as a PC or a tablet. Overall, the efficacy and the utility of VR is well recognized, especially when applied to neuromotor rehabilitation, as it allows to provide fully controllable and personalized simulated real-life environments that gives to the patient the opportunity to exercise safely and to increase the motivation and compliance to the treatment [17].

Despite the several advantages that both robotics and VR offer in rehabilitation programs, considering the perspective of the patient when using the device is essential to guarantee adequate engagement and adherence to treatment. For this reason, the introduction of technological devices in rehabilitation programs has raised the issue of usability. Differently from the concept of feasibility, which is defined as the extent to which a new treatment or innovation can be successfully used or carried out within a given population or setting, usability refers to the patient’s perception and ability to use a system to achieve goals effectively, efficiently, and satisfactorily [18]. According to Nielsen [19], usability may be explained by five attributes, namely learnability, efficiency, memorability, error rate and recovery, and satisfaction. Accordingly, a patient that considers a device as usable presumably also reports a positive perception as concerns: the ease of learning the functionality and behaviour of the system, the effort made to reach the goal, the ease of remembering the system functionality for any further use, the system capability to support and to let easily recover in case of errors during the use, and the pleasantness of the system design. Therefore, evaluating such aspects provides crucial insights into the perceived acceptability and usefulness of the devices, allowing consequently to understand how to improve patient’s motivation during the therapy. As underscored by prior works, motivation plays a pivotal role during rehabilitation program, as it contributes to make the patient feel competent and satisfied [20]. Moreover, patient’s satisfaction with the treatment was found to be associated with stronger therapy compliance [21]. As a result, both factors may be considered key aspects for therapy efficacy. Furthermore, when we aim at patient’s perception of the usability of the devices, it is important to consider the context in which the devices are used. Since, most of the time, the use of the technology during the rehabilitation is supported or mediated by physiotherapists, it is crucial to also elicit their perception, as their success expectations and views on the technological devices may be transferred to the patient [22].

Although the term usability is frequently used, it is defined by both the research community and standard organizations inconsistently [23]. Beside the absence of a clear consensus, usability is also recognized as a construct focused more on the task and less on the experience [24]. For these reasons, research on the usability of robotic and VR devices applied to rehabilitation has explored the role of user experience too, providing so far informative, but contradictory, findings on deeper facets like emotions and affective reactions toward the use of the technology [20]. Therefore, exploring usability and user experience in technology-assisted rehabilitation programs should be of paramount concern, as it would help, on the one hand, to strengthen a recovery methodology that has already shown its efficacy [25] and, on the other hand, it would provide further and informative insight into the perceived evaluation of the specific device implemented. Accordingly, it must be acknowledged that, despite robotic and VR devices have so far shared high technological impact, they basically differ from a technical point of view and for how the user interact with them, ultimately affecting usability and user experience evaluation. For this reason, both technological device typologies were included in the present work specifically with the aim to highlight their impact whether they are implemented in combination or independently. Particularly, this choice may elicit a deeper understanding of the strengths and limitations of the devices described, including their differences. Moreover, in a broader perspective, alongside the well-known increase of life expectancy and, thus, of morbidity, multi-morbidity, and disability [26], providing clear device-specific guidelines along with optimal and customized recovery is increasingly needed [27].

Following this line, the present study aimed at systematically reviewing the literature concerning the evaluation of the usability of technological devices, namely robotics and VR, implemented in combination or independently in the neuromotor rehabilitation context, considering both patients’ and healthcare professionals’ perspectives. To the best of our knowledge, a systematic overview on this topic has not been provided yet.

## Methods

A priori search on registered or ongoing similar contributions was conducted through the International Prospective Register of Systematic Reviews (PROSPERO). The register provided no results and, thus, the systematic review protocol was registered (ref. CRD42021224141). The current work is part of a broader project called PHTinRehab Study (Perception of High Technology in Rehabilitation: a prospective real-life Study on usability, effectiveness, and health-related quality of life*)* approved by the Ethics Committee of ICS Maugeri—Institute of Montescano (February 2021, protocol n. 2517CE).

### Search strategy and selection of the studies

The review was conducted and reported following the Preferred Reporting Items for Systematic Reviews and Meta-Analyses (PRISMA) guidelines [28]. Electronic searches of PubMed, Medline, Scopus, Web of Science, CINAHL, and PsychINFO were performed on 1 March 2021. The following search query was applied for all databases: ((rehabilitation) AND (robot* OR "virtual reality" OR tech*)) AND (usability). Although the current study aimed at identifying published researches specifically on neuromotor rehabilitation, the general term “rehabilitation” was preferred to ensure a full retrieval despite its applicability to different fields (e.g., neuropsychology, psychiatry). Moreover, in the absence of a clear consensus on the terminology of the technological devices, the word “tech*” was specified besides “robot*” and “virtual reality”. This choice was also driven by the intention to retrieve studies that also included wearable tools (e.g., body-mounted sensors) and/or m/eHealth technologies (e.g., smartphone) in combination to robotics and/or VR during the rehabilitation program. Furthermore, to better optimize and refine the identification of the studies according to the eligibility criteria (Table 1), mutual filters (i.e., year of publication timespan, article language, and document typology) were applied for each electronic database consistently. A reference management and bibliography-creating software (EndNote Web) was implemented during the review process.

**Table 1:** Eligibility criteria

**Inclusion criteria**
• Year of publication timespan: 2000 up to date• Full-text articles published in English in a peer-reviewed journal• Quantitative and qualitative studies• Adult patients undergoing technological neuromotor rehabilitation and/or healthcare professionals• Usability evaluation of the technological devices
**Exclusion criteria**
• Patients undergoing not strictly neuromotor rehabilitation (e.g., cognitive rehabilitation)• Healthy participants or patients suffering from psychiatric disorders• Studies on neuromotor rehabilitation with wearable devices and/or m/eHealth tools exclusively• Conference papers, proceedings, study protocols, commentaries, editorials, position papers, reviews

### Quality assessment

To assess the methodological quality of the included studies, the McMaster Critical Review Forms for quantitative and qualitative research [29, 30], which include guidelines for interpreting the criteria [31, 32] to facilitate inter-rater reliability, were used. Since these tools provide a narrative assessment only, the scoring criteria for the guidelines developed by Imms [33] were applied (Table 2). Accordingly, quantitative researches were scored on a checklist of three criteria: sample, measure, and analysis. Qualitative studies were rated on four criteria based on trustworthiness: credibility, transferability, dependability, and confirmability. For each criterion, a score of one (no evidence of study meeting criterion), two (some evidence or unclear reporting) or three (evidence of study meeting criterion) was assigned.

**Table 2:** Risk of bias and quality assessment criteria

Research design	Criteria ^a^	Satisfied if
Qualitative	Credibility	1.Collection of data over a prolonged period and from a range of participants 2.Use of a variety of methods to gather data 3.Use of a reflective approach through keeping a journal of reflections, biases, or preconceptions and ideas 4.Triangulation used to enhance trustworthiness through multiple sources and perspectives to reduce systematic bias. Main types of triangulation are by: sources (people, resources); methods (interviews, observation, focus groups); researchers (team of researchers versus single researchers); or theories (team bring different perspectives to research question) 5.Member checking
	Transferability	1.Can the findings be transferred to other situations? 2.Are the participants and settings described in enough detail to allow for comparisons with your populations of interest? 3.Are there concepts developed that might apply to your clients and their contexts? 4.Were there adequate (thick) descriptions of sample and setting?
	Dependability	1.Is there consistency between the data and the findings? 2.Is there a clear explanation of the process of research including methods of data collection, analysis and interpretation often indicated by evidence of an audit trail or peer review? 3.An audit trail described the decision points made throughout the research process
	Confirmability	1.What strategies were used to limit bias in the research, specifically the neutrality of the data not the researcher? For example, was the researcher reflective and did they keep a reflective journal, peer review such as asking a colleague to audit the decision points throughout the process (peer audit) and checking with expert colleagues about ideas and interpretation of data, checking with participants (participant audit) about ideas and interpretation of data and having a team of researchers
Quantitative	Sample	1.Sample is representative 2.Selection bias reduced: population based/representative/convenient 3.Size of study in relation to design and question (power) 4.Clearly described participant characteristics
	Measure	1.Measure is valid for purpose and reliable 2.Measurement bias is reduced: validity of tools for purpose/reliability of tool/recall/memory
	Analysis	1.Analyses are appropriate to the research question and outcome measure 2.Statistical significance reported 3.Point estimates and variability provided and clinical importance discussed

^a^ A rating of one (no evidence of study meeting criterion), two (some evidence or unclear reporting) or three (evidence of study meeting criterion) was used to rate each criterion

The included studies were assessed by two researchers working independently and any discrepancies were resolved by discussion until a consensus was reached. Recognising that studies rated as lower methodological quality can still provide useful insights based on the data [34], all studies were included regardless of assessment results. Though, study quality was considered in the interpretation of the results.

### Data extraction and synthesis

Eligibility criteria were discussed and accepted after authors’ full consensus. Thereafter, a progression exclusion of the non-eligible records was performed starting from the title, then the abstract, and finally the full-text. The reviewers conducted the entire process by working independently. To solve the disagreements, periodically planned discussions including all authors were carried out. Each identified article was screened multiple times to increase familiarity and obtain a thorough understanding of the study aim, methods, intervention, and outcomes. A wide range of data was extracted and collected in a structured table that, due to its extent, is provided as supplementary material. This includes: author(s), year of publication, authors’ nation, the rank of the nation according to the Human Development Index (HDI), which is a composite index measuring the average achievement in three basic dimensions of human development, namely ‘long and healthy life’ (life expectance at birth), ‘knowledge’ (expected years of schooling and mean years of schooling), and ‘a decent standard of living’ (Gross National Income per capita) [35], the research group’s profession specialty field, study design (follow-up presence and duration, if pilot study, if multicentre, if real-life, any fundings), characteristics of the patients (i.e., inpatients or outpatients, disease, sample size, age, and ethnicity) and of the healthcare professionals involved (i.e., specialty, sample size, age, and ethnicity), the study purpose, the name and a brief description of the technological devices, the level of immersion (in case of VR devices), robot typology (i.e., exoskeleton, end-effector, soft-robot), intervention characteristics (i.e., overall duration, number of sessions, session duration), usability factors investigated, measures (quantitative and qualitative), and main results (i.e., devices strengths and limitations) divided by patients and healthcare professionals. Due to the varying design characteristics of the included studies, both quantitative and qualitative results were analyzed using narrative [36–38] rather than statistical methods. The main findings were discussed and synthetized descriptively.

## Results

### Flow of studies through the review

**Fig. 1:**
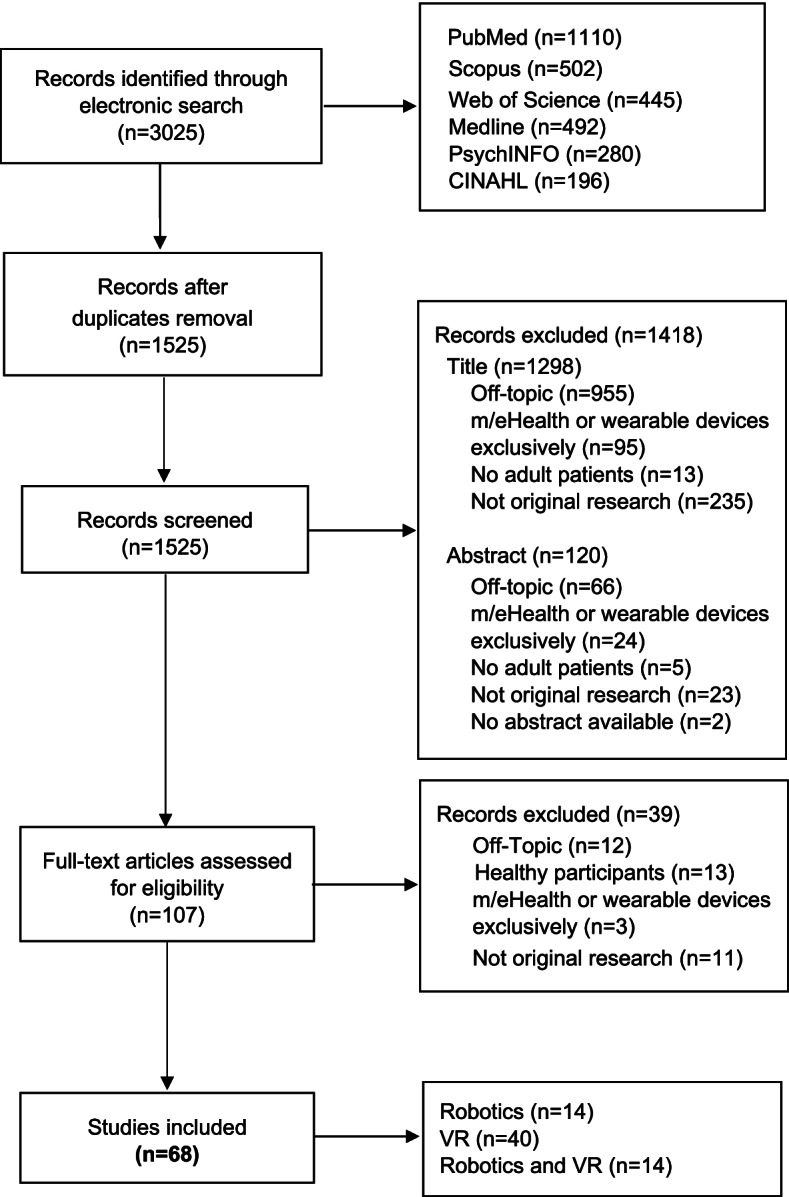
Flow of studies through the review

By initial electronic search 3025 records were retrieved. After duplicates removal, 1525 studies were identified and screened by title and abstract. A total of 1418 studies were excluded and the remaining 107 were assessed for eligibility. Of these, 68 met all inclusion criteria. No additional records were identified by hand searching. Details on study selection and reasons for exclusion are outlined in the flow diagram (Fig. 1). Most of the excluded records were labelled as off-topic (*n *= 1033), as they were not strictly focused on technological neuromotor rehabilitation (e.g., conventional rehabilitation exclusively, cognitive rehabilitation, technological-based interventions aimed at self-care and healthy behaviours promotion/monitoring), others were excluded because considered m/eHealth and/or wearable tools exclusively (*n* = 122).

### Characteristics of the included studies

The full report of the information of the included studies is presented in the synoptic table (Additional File 1: Appendix 1) [39–106].

### Quality

Forty-seven articles used quantitative methods, and 18 studies adopted a mixed approach. Four [66, 69, 76, 105] of these 65 studies provided evidence to satisfy all three criteria for quantitative studies. Thirty-two articles [47, 52–56, 58, 62–65, 67, 68, 71, 72, 74, 75, 77–79, 83, 88, 90, 91, 96–98, 100–103, 106] satisfied two criteria with some evidence of meeting the third. Of the 18 mixed-methods studies, one article [69] satisfied three criteria for qualitative studies with some evidence toward the fourth and five studies [73, 77, 83, 87, 95] satisfied at least one criterion.

Three studies [86, 92, 99] used qualitative methods exclusively. All demonstrated at least some evidence of trustworthiness. In particular, one article [86] satisfied two criteria, and one study [92] met one criterion.

### Design

Tables 3 and 4 summarize the main characteristics of the final studies, including the information on the study design. Most (69.1%) were published in the last 5 years. The most frequent contribution was from research groups from the USA (31.0%) and belonging to the field of physiatry (57.4%) and biomedical engineering (35.3%). Despite this, a heterogeneity of professional contributions was observed denoting a multidisciplinary approach in the study of usability. As for the study design, the majority were feasibility/usability studies (41.2%), which provided a detailed description of the devices and reported their clinical applicability and perceived usability. Only the 11.8% included follow-ups, which ranged from one to three months. Moreover, the majority were supported by fundings (61.7%).

**Table 3:** Main characteristics of the included studies (*n* = 68)

Year of publication	*n* (%)	Nation	HDI ^a^ (ranking)	*n* (%^b^)	Research group specialty field	*n* (%^b^)
2016–2021	47 (69.1)	USA	0.926 (17)	21 (31.0)	Medicine and health sciences	
2011–2015	15 (22.0)	Netherlands	0.944 (8)	11 (16.2)	Physiatry	39 (57.4)
2006–2010	5 (7.4)	Spain	0.904 (25)	11 (16.2)	Neurology	14 (20.5)
2000–2005	1 (1.5)	Italy	0.982 (29)	9 (13.2)	Neuroscience	9 (13.2)
		Switzerland	0.955 (2)	8 (11.8)	Occupational Therapy	9 (13.2)
		South Korea	0.916 (23)	6 (8.8)	Psychology	4 (5.9)
		Canada	0.929 (16)	5 (7.4)	Physiopathology	3 (4.4)
		Australia	0.944 (8)	5 (7.4)	Orthopaedics	2 (2.9)
		UK	0.932 (13)	4 (5.9)	Geriatrics	2 (29)
		Sweden	0.945 (7)	4 (5.9)	Telemedicine	2 (2.9)
		Saudi Arabia	0.854 (40)	3 (4.4)	Public Health	1 (1.5)
		Taiwan	-	3 (4.4)		
		Germany	0.957 (6)	2 (2.9)	Engineering	
		Israel	0.919 (19)	2 (2.9)	Biomedical Engineering	24 (35.3)
		Mexico	0.779 (74)	2 (2.9)	Computer Engineering	11 (16.2)
		Portugal	0.864 (38)	1 (1.5)	Mechanical Engineering	7 (10.3)
		New Zealand	0.931 (14)	1 (1.5)	Electrical Engineering	2 (2.9)
		Austria	0.922 (18)	1 (1.5)		
		India	0.645 (131)	1 (1.5)	Other sciences	
		France	0.901 (26)	1 (1.5)	Computer Science	11 (16.2)
		Japan	0.919 (19)	1 (1.5)	Informatics	4 (5.9)
		Ireland	0.955 (2)	1 (1.5)	Physics	3 (4.4)
		Paraguay	0.728 (103)	1 (1.5)		
		Poland	0.880 (35)	1 (1.5)		
		Belgium	0.931 (14)	1 (1.5)		
		China	0.761 (85)	1 (1.5)		

^a^ HDI index is based on 3 dimensions: (a) Life expectance at birth; (b) Expected years of schooling and mean years of schooling; (c) Gross National Income per capita (United Nations Development Programme, http://hdr.undp.org/en. Accessed 1 April 2021)

^b^ Non-cumulative percentages

**Table 4:** Main study design characteristics of the included studies (*n* = 68)

Study design	*n* (%^a^)	Follow-up	*n* (%)	Funding(s)	*n* (%)	Multicenter	*n* (%)	Pilot Study	*n* (%)
Feasibility/usability study	28 (41.2)	Yes ^b^	8 (11.8)	Yes	42 (61.7)	Yes	13 (19.1)	Yes	25 (36.8)
Observational study	18 (26.5)	No	60 (88.2)	No	26 (38.3)	No	55 (80.9)	No	43 (63.2)
RCT	9 (13.2)								
Case study	6 (8.8)								
Clinical trial	4 (5.9)								
Quasi-experimental study	3 (4.4)								
Experimental study	1 (1.5)								

^a^ Non-cumulative percentages ^b^ Follow-up range: 1–3 months

### Patients and healthcare professionals involved

As for the participants (Table 5), the total number of patients of the included studies was 1464 and the sample sizes varied widely with a minimum of two patients and a maximum of 157. Notably, most of the studies (86.2%) presented limited sample sizes including less than 30 participants. The age ranged from 18 to 91 years and only one study [87] reported the ethnicity of the patients. As regards to healthcare professionals enrolled, the total number was 72, with the physiotherapists as the most recruited category (88.9%). The sample sizes varied from two to 20 participants.

**Table 5:** Main participants’ characteristics of the included studies (patients, *n* = 65; healthcare professionals, *n* = 18)

Patients	*n* (%)	Disease	*n* (%^c^)	Healthcare professionals	*n* (%^c^)
Inpatients	19 (29.2)	Stroke	45 (69.2)	Physiotherapists	16 (88.9)
Outpatients	21 (32.3)	Musculoskeletal disorders ^a^	12 (18.5)	Occupational therapists	4 (22.2)
Not defined	25 (38.5)	Multiple Sclerosis	6 (9.2)	Physiatrists	3 (16.7)
		Traumatic Brain Injury	6 (9.2)		
		Spinal Cord Injury	4 (6.1)		
		Parkinson’s disease	3 (4.6)		
		Other neurological diseases ^b^	7 (10.8)		
		Geriatric	1 (1.5)		
		Cardiopulmonary	1 (1.5)		

^a^ Rheumatoid arthritis, Osteoarthritis, Carpal tunnel syndrome, Hand disability, Chronic pain, Unicompartmental and Total knee arthroplasty ^b^ Spinal stenosis, Guillain-barré syndrome, Vestibular disorders ^c^ Non-cumulative percentages

### Devices adopted and rehabilitation sessions’ characteristics

Table 6 shows the main characteristics of the technology used and of the rehabilitation program. Overall, the 58.8% of the studies implemented VR systems and the 20.6% used robotic devices, while the remaining 20.6% used both. VR systems were mostly non-immersive (77.7%) and provided a wide range of activities such as VR-based treadmill training for lower extremities functionality or exercises in reaching and grasping virtual objects for upper limbs mobility and manual dexterity. The studies describing robot-assisted interventions mainly used exoskeleton devices (60.7%) and proposed both active and passive robot-assisted gait training as well as re-learning activities for the arms and upper extremities through kinematics exercises from complete movement guidance to the absence of support and with the integration of game-based VR environments to interact with during the therapy session. The mean duration of the technological rehabilitation along with the mean of the sessions and duration were extracted. VR-based therapies reported wider ranges than those assisted by robotics. Specifically, the duration of the interventions based on the combination of both technologies lasted from one to 9 weeks with a total number of sessions ranging from one to 36 (session duration range: 10–90 min).

**Table 6:** Main characteristics of the technological devices and of the interventions of the included studies (*n* = 68)

	**VR**	**Robotics**	**VR and Robotics**
Included studies, *n* (%)	40 (58.8)	14 (20.6)	14 (20.6)
VR level of immersion, *n* (%)			
Non-immersive	28 (70.0)	-	14 (100.0) ^a^
Semi-immersive	6 (15.0)	-	-
Fully-immersive	6 (15.0)	-	1 (7.1) ^a^
Robot typology, *n* (%)			
Exoskeleton	-	10 (71.4)	7 (50.0)
End-effector	-	2 (14.3)	4 (28.6)
Soft-robotics	-	2 (14.3)	3 (21.4)
Intervention, mean ± SD (range)			
Overall Duration (weeks)	4.5 ± 2.9 (1–12)	5.0 ± 2.3 (1–9)	5.7 ± 1.8 (4–8)
n. of sessions	11.4 ± 18.7 (1–84)	8.9 ± 7.8 (1–20)	13.8 ± 14.5 (1–36)
Session duration (min)	33.2 ± 33.7 (3–180)	55.0 ± 24.8 (10–90)	40.0 ± 25.0 (10–90)

^a^ Non-cumulative percentages

### Usability

**Fig. 2:**
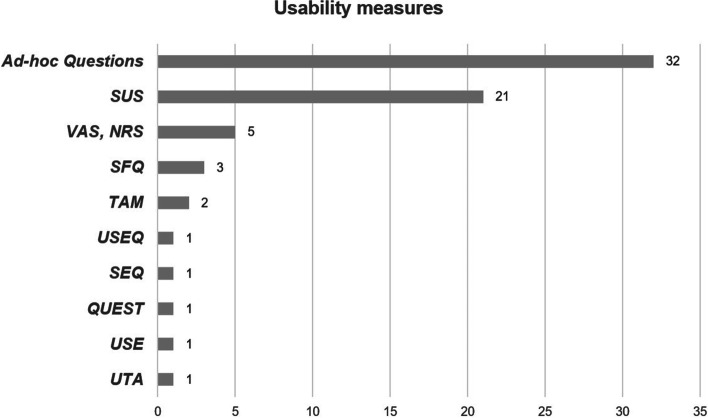
Quantitative usability measures of the included studies. SUS, System Usability Scale; VAS, Visual Analogue Scale; NRS, Numerical Rating Scale; SFQ, Short Feedback Questionnaire; TAM, Technology Acceptance Model Questionnaire; USEQ, User Satisfaction Evaluation Questionnaire; SEQ, Suitability Evaluation Questionnaire; QUEST, Quebec User Evaluation of Satisfaction with Assistive Technology 2.0; USE, Usefulness, Satisfaction, Ease of Use Questionnaire; UTA, Users’ Technology Acceptance Questionnaire

To evaluate usability, 47 studies (69.1%) used quantitative measures only. Three studies (4.4%) conducted a qualitative evaluation exclusively, while 18 (26.5%) adopted a mixed-methods approach. The System Usability Scale (SUS) [107] was the most frequently administered scale (47.7%). Also, ad-hoc questions (49.2%), and Visual Analogue Scales (VAS) and Numerical Rating Scales (NRS) (7.7%) were implemented (Fig. 2). Of the qualitative and mixed-methods studies, open-ended questions (42.9%) were used and interviews with different structure (38.1%) and focus groups (19.0%) were conducted. Multiple usability-related factors were assessed (Table 7). Overall, the most frequent were ease-of-use (82.4%), learnability (52.9%), and satisfaction (29.4%). Others were more strictly related to the patients’ experience of use of the technological devices, namely motivation (36.7%), enjoyment (22.1%), adverse effects (13.2%), and engagement (8.8%).

**Table 7:** Usability and user experience parameters divided for device typology in the included studies (*n* = 68)

VR	*n* (%^a^)	Robotics	*n* (%^a^)	VR and Robotics	n (%^a^)
Ease-of-use	33 (82.5)	Ease-of-use	11 (78.6)	Ease-of-use	12 (85.7)
Learnability	22 (55.0)	Satisfaction	8 (57.1)	Learnability	9 (64.3)
Motivation	18 (45.0)	Effectiveness	6 (42.9)	Motivation	5 (35.7)
Enjoyment	13 (32.5)	Learnability	5 (35.7)	Acceptability	3 (21.4)
Satisfaction	10 (25.0)	Comfort	3 (21.4)	Safety	2 (14.2)
Acceptability	8 (20.0)	Acceptability	3 (21.4)	Satisfaction	2 (14.2)
Adverse effects	8 (20.0)	Safety	2 (14.2)	Engagement	2 (14.2)
Sense of presence	6 (15.0)				
Usefulness	5 (12.5)				
Engagement	5 (12.5)				

^a^ Non-cumulative percentages

### Virtual Reality

Overall, VR devices were rated as having good usability. Patients referred good acceptability regardless the different levels of immersion. Most participated in non-immersive VR-based therapy [40, 44–46, 48, 50, 52, 53, 57–62, 64–73, 75–77], whereas fully-immersive devices (i.e., HMD) were used in six studies [39, 42, 43, 54, 63, 78]. Semi-immersive VR (i.e., Kinect with large TV or projector screens) was tested in five studies [47, 49, 51, 55, 57, 74]. Moreover, some studies combined the use of VR with devices for conventional rehabilitation as treadmill [55], force plate [52], springboard [62] and bicycle [74]. Satisfactory scores on the devices ease-of-use were observed mostly [39, 40, 43, 45–48, 50–59, 62, 63, 65, 67–72, 74–78] along with the levels of satisfaction [45, 48, 49, 54–56, 58, 59, 61, 63, 70] and learnability [39–43, 46–48, 51, 55–57, 62, 67, 69, 71, 72, 74–78]. Concerning the user experience, the majority found the rehabilitation as motivating and engaging [40, 42–44, 47, 49, 50, 53, 55, 58, 60, 63, 67, 69, 70, 73–76], reported high levels of enjoyment [40, 45, 48, 55, 59, 64, 66, 68, 69, 71–73, 76], and referred none to few adverse effects (e.g., nausea, disorientation, dizziness) [43, 50, 51, 54, 55, 57, 63–66, 68, 71–73, 76, 78]. Some patients agreed with the efficacy of the devices perceiving physical health benefits [39, 58, 61, 72, 75] and expressed the intention of future use in both clinical and home settings [53, 57–59, 64, 69, 75]. However, although the devices were on average considered easy to learn and to understand, learnability was mentioned as a factor affecting patients’ performance during the initial phases of the therapy. Accordingly, brief tutorials and more trainings before rehabilitation sessions were suggested [39, 50, 60, 69]. Further, some patients felt that more mental effort was required than usual while performing VR therapies, leading in some cases to experience high cognitive load and consequent loss of concentration [40, 60, 64, 75, 77]. Finally, some technical issues (e.g., size of the screen, device comfort, game design, feedback quality) were raised [42, 49, 50, 52, 53, 56, 57, 64, 70]. These led patients to have difficulties in terms of interactivity with the VE [59, 61, 68] and consequently contributed to the need for more technical support from the therapist [52, 53, 77].

Healthcare professionals provided overall positive feedback on devices ease-of-use, comfort, learnability, and usefulness along with perceived efficacy [39, 40, 48, 49, 53, 60, 62]. Notably, they appreciated the potential for customized therapy sessions and the benefits deriving from the patient’s performance monitoring system [40–43, 64, 73]. Despite the perceived high applicability to clinical settings, healthcare professionals raised some limitations, too. The role of learnability in affecting initial patient’s performance was confirmed. Consistently, increasing the number of training sessions or the introduction of tutorials before starting the therapy were recommended [39, 41, 60]. Furthermore, some remarked that the complexity of the VE graphic design may represent a distracting factor leading to weaken patient’s compliance [60]. Finally, the importance of system adaptability to patient’s abilities to ensure therapy effectiveness was underscored, too [41, 60].

### Robotics

Generally, robotic devices were perceived as usable. Patients well accepted their implementation to the rehabilitation programs. Most underwent exoskeleton-assisted therapies [80–82, 84, 85, 87, 89–92], whereas others used end-effector devices [79, 83]. Two studies evaluated the use of soft-robots [86, 88]. Moreover, of the studies including exoskeletons, two added the use of devices for conventional rehabilitation, namely a walker and bilateral crutches [84] and a treadmill [90]. Overall, patients rated positively devices ease-of-use [79–81, 83, 85–88, 90–92] and learnability [79, 81, 82, 88, 91]. They considered themselves satisfied with the training program [79, 82, 84, 85, 87, 89–91] and found the treatment motivating and enjoyable [80, 82, 83, 85, 89, 90]. Furthermore, health benefits concerning the lasting effects on mobility, balance, gait, independence, and psychosocial well-being were reported [82, 83, 86, 87, 89, 92]. Accordingly, some patients suggested devices implementation in the future [82, 87, 88]. Nevertheless, some technical limitations were raised. Mainly, issues concerning devices design were observed, as low comfort and manageability, and difficulties in donning and controlling the device autonomously [81, 83–87, 92]. Moreover, technical issues like the mechanical resistance of the device were mentioned as difficult to overcome and, in some cases, physically straining [81]. In conclusion, suggestions to improve the adaptability (e.g., transportability, ability to walk up and down stairs, surface adaptability) were reported to ensure the implementation also outside the therapy [87, 92].

Concerning healthcare professionals’ perspective, the devices were judged as having wide applicability and potential for increased treatment intensity and safety with the possibility to quantitatively monitor patients’ parameters throughout the entire recovery process [79, 85, 90, 92]. In contrast, some technical and design issues were reported. Some expressed the need for an effortless, time-saving, and flexible system to ensure optimal clinical applicability [85, 90, 92]. Accordingly, stronger collaborations with developers and final users were considered essential [92]. Finally, patient’s cognitive and communication deficits were evidenced as potential barriers in systems use affecting, in turn, therapy efficacy [92].

### Virtual Reality and Robotics

Some of the studies presented the usability evaluation of combined VR and robotics. The majority implemented exoskeleton devices [93, 94, 97–99, 103, 104], whereas others used end-effector robots [94, 95, 104, 105]. Moreover, three studies described the use of soft-robots [100–102]. The combination with the VR exclusively consisted of non-immersive systems (e.g., monitor, PC), except for one study, which interestingly included both non-immersive and fully-immersive (i.e., CAVE) technologies [97]. Combining the two different device typologies resulted in satisfactory usability rates. Most of the studies reported the rehabilitation program to be motivating, pleasant, and meaningful [94, 98, 99, 101–103] along with positive patients’ feedback on the devices ease-of-use [93, 95–98, 100, 102, 104–106], learnability [93, 96–98, 100–102, 104, 105] and satisfaction [95, 103]. One study highlighted patients’ perceived effectiveness of the device, which contributed to improve their quality of life [97]. In general, no specific technical support was expressed, except for one study [96], which also pointed out patients’ desire to use the device again in the future, though [96]. Some limitations were remarked too. These included the robotic active assistance of some devices, which was considered in some cases discordant with patient’s intended movement [99]. In some cases, the mechanical complexity of the devices did not allow a fluent control, which in turn affected the interaction with the virtual objects displayed in the VE and led patients to experience some frustration [98].

As for the healthcare professionals, only one study describing the combination of robotics with VR was included [95]. The possibility to associate the robot-assisted therapy with the implementation of software interfaces, which provided visual feedback to the patient and consequently improved treatment motivation and compliance was appreciated. Lastly, among the suggestions are the preference of a wider range of VR games to avoid patient’s interest loss during the session and the integration of more therapy techniques-specific game exercises to maximise rehabilitation efficacy.

## Discussion

The present study aimed at systematically reviewing the current literature on the usability of VR and robotic devices applied to neuromotor rehabilitation. Both patients and healthcare professionals’ perspective on this topic was considered.

From the review process, 68 studies were included and synthetized. Promisingly, the number of studies describing the perceived acceptability of the devices has been increasing sensibly through time. As shown in the current review, in the last two decades most of the studies date back within the last five years, meaning that the growing interest in evaluating the usability issues of VR and robotics in neuromotor rehabilitation is rapid and recent. Most of the included researches reported the application of VR, whereas those describing the use of robotics or their combination represented the minority. The absence of an equal distribution in terms of studies numerousness may be attributable to the economic considerations toward the use of robot technology. Indeed, robot-assisted rehabilitation requires higher levels of investments, and its maintenance and routine operation are recognized to be relatively costly. This consequently leads to a growing need for a cost-effectiveness analysis [108], which for the implementation of VR systems has by contrast received a clearer consensus, as it was evidenced a wider accessible strategy that provides high recovery benefits along with lower costs. Purposely, off-the-shelf and commercially available video gaming systems have been already proposed and adapted for use in VR rehabilitation showing satisfactory results [109].

Despite these differences, the implementation of robotics and VR in rehabilitation programs entails higher costs when compared to conventional treatment, making the research on this topic more expensive. Accordingly, the majority of the studies included in the present review received a financial support whether they were pilot feasibility researches or clinical trials. Moreover, these studies were essentially carried out by research groups from high-ranked countries according to the HDI [35] with evidently more economic resources to be invested in this type of studies.

Despite the resources, it should be pointed out that one study out of three was pilot and lacked further evidence, though. Notably, most explored the benefits and the limitations of the technological devices and gave insight into feasibility and formal testing, but almost none moved from intervention efficacy trials to scale-up evaluations in real-life settings. The transition from efficacy to effectiveness represents a still open challenge that future research on this topic should consider net of the issues related to the complexity and the cost of the devices, as well as the characteristics of the population involved [110]. Following this line, addressing these issues and including questions related to the needs and the perception of the end-user from the earlier intervention phases should be of paramount concern and would have the potential to facilitate the scaling-up process [111]. In support of this, longitudinal study designs may also contribute to provide clearer evidence on the effectiveness of technological rehabilitation, as follow-up evaluations would shed light on the effects on patients over time. Most of the synthetized studies did not include follow-ups, and those reporting were not over three months. Future research should take into account this aspect and include larger follow-ups to better clarify the perceived effectiveness and the resonance of VR and robotics in real-life.

A heterogeneity of authors contributions emerged within research groups including different professional categories. Accordingly, not only healthcare professionals contributed, but also experts from different fields of engineering. This reflects the current trend of a constantly evolving healthcare system that encompasses differentiated resources designed to innovation and progress in recovery processes. However, although usability and the experience of use of the devices are mainly recognized to be psychological-related constructs, a paucity of studies including a contribution from the psychological field was observed. In this vein, future studies interested in such aspects should involve more specialised professional figures to discuss the findings more appropriately.

Further heterogeneity was observed concerning the participants characteristics, VR systems and robot typologies used. The included studies involved patients with different neurological and musculoskeletal pathologies and healthcare professionals of different occupations. Although most of the patients suffered from stroke, this main prevalence is not surprising if we consider that nowadays the estimation of stroke cases is 200 per 100.000 inhabitants (70% over 64 years) with an increasing global burden in both sexes [112, 113]. Additionally, a wide range in terms of sample size was observed for both groups of participants. Despite this, most of the articles involved less than 30 patients and less than 10 healthcare professionals. In future studies, larger sample sizes are recommended. Lastly, of the socio-demographic data extracted, only one study reported the ethnicity of the samples. As suggested in a prior work [114], future clinical research should be encouraged to report participants’ ethnicity diversity for multiple reasons, including increased results generalisability. Concerning the technological devices used, different VR systems with different levels of immersion and various robot typologies were implemented. Regarding specifically the VR, the level of immersion provided by the systems implemented was not always clearly defined within the studies included. This reflects, at least in the field of neuromotor rehabilitation, the absence of a clear consensus on VR immersion classification, making it difficult to navigate the literature and generalize the effectiveness and feasibility of specific rehabilitation systems [115]. Following this line, future studies in this field should adopt a shared framework in order to explain and better clarify the key characteristics of VR and their clinical implications when implementing a specific device. Accordingly, immersion, sense of presence, and interactivity should be considered as key aspects existing on a continuum and, consequently, should be targeted adopting a framework able to address their complexity and the extent of the underlying interacting elements [116, 117]. Overall, the heterogeneity observed in terms of device typology implementation may be ascribable to an absence of clear guidelines orienting the choice of a certain device in relation to specific therapeutic goals and, also, to a widespread difficulty in dealing with the devices upgrade because of the costs and design complexity. Moreover, the ever-growing number of companies providing new devices that require initial testing may represents a possible further explanation.

**Fig. 3:**
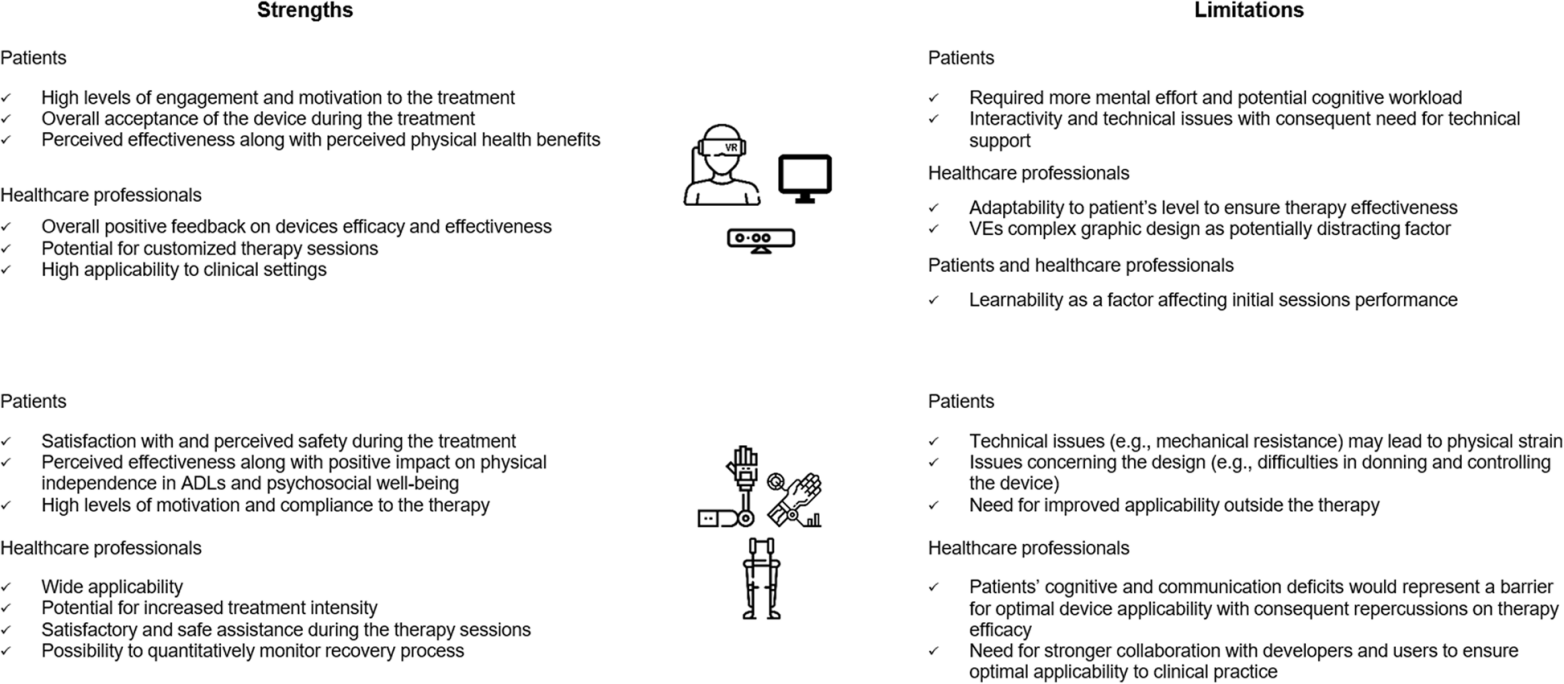
Overall virtual reality and robotic devices strengths and limitations according to patients’ and healthcare professionals’ perspective

Usability evaluation was mixed-methods and focused on multiple factors that varied depending on the device typology. Mutual factors included the level of ease-of-use, the degree of satisfaction, and learnability. Others like the level of enjoyment, motivation, and sense of presence were more related to the interaction with VEs, while parameters such as the perceived safety, the level of comfort, and the perceived effectiveness were mostly observed in robot-assisted rehabilitation. Generally, the use of VR [39–78] and robotics [79–92], as well as their combination [93–106], provided informative insights into strengths and limitations of their deployment (Fig. 3). It must be noted that the number of studies describing the usability of combined robotic and VR devices was not sufficient to provide robust inferences. Accordingly, a deeper analysis of the independent implementation of the two devices was preferred. This choice was also based on the fact that, as previously explained, although the combination of robotic and VR devices has so far provided promising evidence regarding the clinical impact, they remain different technologies as for technical and interactivity issues, ultimately needing a differentiated examination on the related experience of use.

As to strengths, patients stated that VR improved their engagement and motivation during the treatment, denoting an overall acceptance of the devices. Besides, positive effects of the devices on their physical health were perceived right after the treatment. Consistently, healthcare professionals provided positive feedback, suggesting the potential of VR for more tailored interventions through the high clinical applicability to different diseases. As for robotics, patients generally felt satisfied with the interventions showing high levels of compliance to the treatment. The assistance of robotics provided lasting effects on their physical health, and a positive impact on the level of independence and on the psychosocial well-being was perceived. Healthcare professionals agreed with the wide applicability of the devices underscoring their potential for increased treatment safety and intensity and the opportunity to quantitatively monitor patient’s recovery process.

Concerning the limits, learnability was cited by both patients and healthcare professionals as a factor affecting initial sessions performance. Some declared that tutorials or training sessions before the treatment would have benefited the use of the devices. Moreover, VR sessions length and the multiple tasks were judged by patients as requiring more mental effort than usual leading, in some cases, to cognitive workload. Also, technical complexity of the system was perceived as affecting the level of interactivity with the VE raising a need for technical support throughout the intervention. Besides, healthcare professionals expressed the need for adaptable parameters to patient’s level and for lower complexity of the VE graphic design to avoid patient’s distraction. Regarding robotics, patients complained about the mechanical resistance or the low comfort of some systems, which made the treatment, in some cases, physically straining. Moreover, a need for a wider applicability across the continuum of care was expressed along with the desire to use the devices outside the clinical setting. Lastly, patients’ cognitive and communication deficits were considered as potential barriers for device use, potentially affecting therapy efficacy. Overall, devices technical complexity and learnability seemed to represent a relevant limit.

According to the above reported comments, to ensure optimal clinical applicability, healthcare professionals suggested a stronger collaboration with developers and end-users. Besides the well-known utility and efficacy, the introduction and the deployment of technologies to rehabilitation programs may not always result easy, especially for the healthcare professionals whose perspective on their work modalities may change requiring time to adapt. Future studies should address this aspect in real-life settings to better respond to the usability issues of such technologies. From the earlier phases of the intervention, making the devices easier to learn and easier to use would help the end-user to optimally benefit from technological rehabilitation, so as to maximise its effectiveness.

## Supplementary Information

**Additional file 1.**

**Additional file 2.**

## Data Availability

Not applicable.

## Abbreviations

VR: Virtual reality
VE: Virtual environment
ADLs: Activities of daily living
HMD: Head-mounted display
CAVE: Cave automatic virtual environment
PRISMA: Preferred reporting items for systematic reviews and meta-analyses
HDI: Human development index
