# Supplementary material for: A systematic review on the usability of robotic and virtual reality devices in neuromotor rehabilitation: patients’ and healthcare professionals’ perspective
Source: BMC Health Serv Res. 2022 Apr 20;22:523. doi: 10.1186/s12913-022-07821-w (PMC9020115; doi:10.1186/s12913-022-07821-w)
Supplement: Supplementary file 2 — Additional file 2. [file 12913_2022_7821_MOESM2_ESM.docx]

**Additional File Legend**

**Additional File 1. Appendix 1:** Summary of the included studies and results from quality assessment
